# Depression, Anxiety, and Cognitive Distortion among College Students: A Cross-Lagged Prospective Network Study during and after the COVID-19 Pandemic

**DOI:** 10.1155/2024/2598864

**Published:** 2024-06-27

**Authors:** Hongyu Zou, Junyao Gao, Wanchun Wu, Wei Zhang, Lijuan Huo

**Affiliations:** ^1^Key Laboratory of Brain, Cognition and Education Sciences, Ministry of Education, South China Normal University, Guangzhou 510631, China; ^2^School of Psychology, South China Normal University, Guangzhou 510631, China; ^3^Institute for Brain Research and Rehabilitation, South China Normal University, Guangzhou 510631, China; ^4^Department of Psychiatry, The Affiliated Brain Hospital of Guangzhou Medical University, Guangzhou 510370, China

## Abstract

**Background:**

The high prevalence of depressive and anxiety symptoms among college students during the COVID-19 pandemic raised serious global public health concerns. Grounded in Beck's cognitive theory, we tested whether cognitive distortion (included form of rumination) influenced the development of depression and anxiety during and after the pandemic.

**Materials and Methods:**

A total of 2,709 college students in southern China completed self-report measures of depression and anxiety as part of a psychological screening questionnaire during the COVID-19 lockdown. Six months later, after lockdown, 689 of these students completed the same questionnaires.

**Results:**

A cross-sectional network analysis showed that relative to their self-reports during the pandemic, college students reported fewer depressive symptoms and higher anxiety symptoms after the pandemic. A cross-lagged network among depression, anxiety, and cognitive distortion exhibited a consistent pattern, with symptom rumination serving as central node. Surprisingly, depression predicted later anxiety, but anxiety did not predict later depression.

**Conclusion:**

Symptoms of depression and anxiety were uniquely related to different cognitive distortions, suggesting different mechanisms of development during the pandemic. Symptom rumination should be considered a key target in cognitive behavioral therapy.

## 1. Introduction

In recent years, college students' depression and anxiety have emerged as major global public health concerns [1]. College students showed a especially high prevalence of both forms of distress during the COVID-19 pandemic. According to a meta-analysis of 706,415 college students, the incidence of depressive symptoms and anxiety symptoms during the pandemic was 39% and 36%, respectively. These rates were considerably higher compared to the estimated prevalence prior to the outbreak [2]. Investigating the factors underlying these forms of distress and identifying critical areas for psychological intervention have long been important topics in psychopathology research.

### 1.1. Literature Review

**Figure 1:**
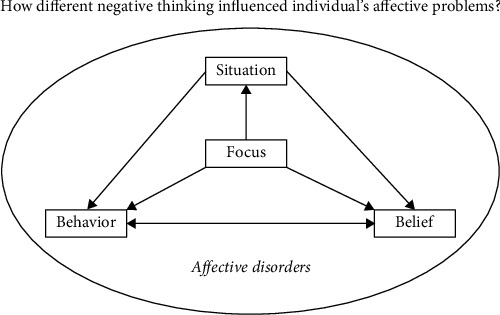
Beck's cognitive theory of affective disorders (adapted from [4]).

People exhibit a range of psychological responses to experiences that increase the risk of depression. These individual differences might explain why an experience would predict depression among some people and not others. Beck's cognitive theory of depression and anxiety proposes that cognitive distortion is an important factor in the occurrence of depression and other affective problems [3]. Other factors that predict depression include situation (events), behaviors, and beliefs (i.e., cognitive distortion). The theory also highlights the unique features of mental disorders that stem from the content of these beliefs (like cognitive distortion).  Figure 1 illustrates Beck's cognitive theory.

Different types of cognitive distortion coincide with depression and anxiety [5, 6, 7] and with the individual symptoms of depression and anxiety is, when compared with boys, exhibited more anxiety symptoms and anxiety sensitivity in 2016. They also reported a significant increase in the mean depression level from 2016 to 2017. Regression analyses revealed the physical concern dimension of anxiety symptoms. In conclusion, intervention through anxiety sensitivity training to reduce somatic concerns and attentional bias modification to increase habitual attention to positive stimuli and to disengage from negative stimuli can reduce anxiety and depression [8, 9]. Investigations of the interrelationships between cognitive distortion and depression and anxiety, and the identification of the key forms of cognitive distortion associated with these symptoms, have implications for preventing and treating depressive and anxiety symptoms in college students. Cognitive behavioral therapy is designed to reduce the cognitive distortion that underlie depression and anxiety [3].

Two types of cognitive distortion appear to be particularly relevant: negative automatic thoughts and negative attentional bias. Negative automatic thoughts are spontaneous pessimistic thoughts in response to unpleasant experiences [10]. A network analysis revealed that the most important node was negative automatic thoughts, which could predict future symptoms of depression and anxiety [11]. Negative automatic thinking is considered a crucial proximal factor in the development of depression and anxiety disorders in cognitive behavioral therapy [3].

Negative attentional bias is evident when an individual consistently contemplates and prioritizes unfavorable information [12]. A longitudinal study showed that individuals who exhibited a tendency to direct their attention toward negative information at the beginning of the study were more likely to experience symptoms of depression and anxiety 1 year later [8]. Beevers et al. [13] showed that it was possible to reduce a negative attention bias using dot detection task training and to effectively reduce specific symptoms of depression in pre–post comparisons [13].

In addition to these examples of cognitive distortion, there is another problematic pattern of thinking, namely, rumination, which is associated with anxiety and depression. Rumination is a cognitive process in which a person repeatedly and passively thinks about negative emotions or events they have experienced [14]. A meta-analysis that included 179 studies and 37 clinical studies reported that rumination could significantly predict the occurrence of depression and anxiety symptoms [15]. Many studies have additionally demonstrated that rumination exerts a significant influence on the onset and maintenance of depression.

Treynor et al. [16] identified three subtypes of rumination, contributing to a more comprehensive understanding of the mechanism through which rumination impacts depression. Different subtypes of rumination exhibit distinct effects on the experience of depression [17]. There is currently a lack of research on the relationship between the three subtypes of cognitive distortion and depression and anxiety from the phenomenological perspective.

Network analysis methods in the study of psychopathology have increased in recent years [18]. This approach relies on the comorbidity hypothesis, which suggests that some symptoms of different disorders are interconnected within the same network. When a certain symptom arises, it will impact the occurrence of another symptom due to the network relationship. Certain psychiatric disorders are considered to be the result of complex interplay between various symptoms [19]. This is unlike traditional perspectives on psychopathology, which posit that mental illness rises from a confluence of underlying factors. The theoretical hypothesis places greater importance on the interplay between various variables, which is in line with the findings of Barabási et al. [20]. Furthermore, the network analysis technique enables the identification of the central nodes within the comorbidity network [21]. These central nodes may be especially relevant in the treatment of psychological disorders.

Currently, the majority of studies employing network analysis techniques to investigate the connection between depressive symptoms, anxiety symptoms, and negative patterns of thinking have used cross-sectional network analysis [22, 23, 24]. However, this methodology is limited to generating correlations, and it lacks the ability to make causal inferences. However, direction of association is crucial in guiding intervention. Hence, the cross-lagged network analysis technique was introduced to establish a directed network that can examine the temporal order of two interconnected variables [25]. The primary objective of this study was to test the longitudinal network association between college students' patterns of negative thinking and their symptoms of depression and anxiety, during and after the COVID-19 pandemic.

### 1.2. Scientific Hypotheses

This study used cross-lagged network analysis to examine college students' psychological functioning during (T1) and after (T2) the COVID-19 pandemic 6 months later. We tested whether there were T1 to T2 changes in the associations among (a) five patterns of problematic thinking (negative automatic thinking, negative attention bias, symptom rumination, brooding, and reflective pondering as examples of rumination), (b) depressive symptoms, and (c) anxiety symptoms. Based on the research literature, we proposed the following hypotheses: (1) the constructed simultaneous network will include edges that are positively and significantly correlated, and (2) the constructed temporal network will show that a reduction from T1 to T2 in any of the five negative cognition patterns will be associated with a decrease from T1 to T2 in anxiety and depressive symptoms.

## 2. Method

### 2.1. Participants and Procedure

The mental health screening for this study was conducted using a cluster sampling method in a university in South China. Participants were college students who were required to be between the ages of 18 and 24 years old. The questionnaires were distributed in person, and participants were asked to complete the questions on either a computer or a mobile phone by scanning a QR code or accessing a provided link (wjx.cn). Upon completion of the questionnaire, the participants were provided a detailed personal mental health report. A total of 2,709 valid questionnaires were collected at Time 1. Some participants who took part in the first questionnaire collection during the COVID-19 were unable to participate again at Time 2 (the time gap was 6 months) because they finished their program in their third year of college. As a result, only 1,745 questionnaires were obtained in the second stage, which comprised newly enrolled individuals. There were 689 participants who completed questionnaires at both time points, and these questionnaires were further screened for validity.

**Figure 2:**
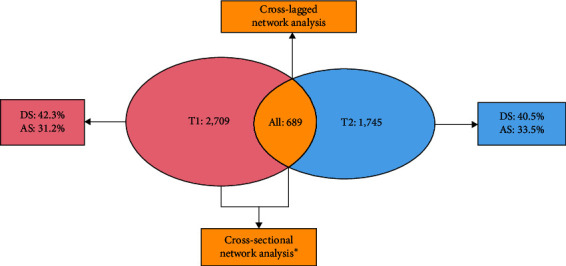
Procedure for enrollment (the circles represent the number of participants in different situations; DS = depression symptoms; AS = anxiety symptoms. *⁣*^*∗*^T2 only included sample data that could match the demographic variables when conducting cross-sectional network analysis because the second batch of data lacked demographic variables).

Several practices were implemented to maximize the validity and reliability of the data. At both time points, questionnaires were excluded if one of the lie detection questions was answered incorrectly, there were one or more than one missing responses, response time was less than 8 min, there was a mismatch between age and grade, there was an outlier score, or any demographic information was missing. The questionnaires obtained at the two time points were matched using codes. There were 689 participants with complete data at both time points.  Figure 2 illustrates the enrollment process.  Table 1 presents the participants' demographic information. All participants have signed the informed consent form online. The study was approved by the Ethics Committee of the School of Psychology, South China Normal University, with the ethics approval number of SCNU-PSY-2022-217.

### 2.2. Measures

#### 2.2.1. The Patient Health Questionnaire (PHQ-9)

The PHQ-9 is a self-report measure of depression with strong psychometric properties [26]. The Chinese version of the PHQ-9 scale has also demonstrated good reliability and validity [27]. We used this scale as a measure of depressive symptoms. The scale consists of nine items (e.g., “feeling tired or having little energy”) that are rated on a four-point Likert scale ranging from 0 (not at all) to 3 (almost every day). A higher total score indicates more frequent depressive symptoms. The Cronbach's alpha for the scale in this study was 0.902 at T1 and 0.918 at T2.

#### 2.2.2. The Generalized Anxiety Disorder Anxiety Scale (GAD-7)

The GAD-7 [28] is a self-report measure of generalized anxiety symptoms. Gong et al. [29] created a Chinese language version of the scale to measure the presence and severity of anxiety symptoms within a Chinese context. The seven items are rated on a four-point Likert scale ranging from 0 (not at all) to 3 (almost every day). Participants are asked how they have felt over the past 2 weeks (e.g., “feeling nervous, anxious, or on edge”). The higher the total score, the worse the anxiety symptoms. The Cronbach's alpha of the scale was 0.931 and 0.940 at T1 and T2, respectively.

#### 2.2.3. The Ruminative Response Scale (RRS)

The RRS was created by Nolen–Hoeksema [14], and a Chinese version of the scale was created by Han and Yang [30]. The scale contains 22 items and has three subdimensions: symptom rumination, brooding, and reflective pondering. These subdimensions reflect individuals' various cognitive responses to depression (e.g., I think, why do I always react this way?) [16]. Each item is rated on a four-point Likert scale, ranging from 1 (almost never) to 4 (almost always). The Cronbach's alphas for the scale at T1 and T2 were 0.947 and 0.977, respectively.

#### 2.2.4. The Attention to Negative Information Scale (ANIS)

The ANIS was used to assess how much people focus on and think about negative information about themselves or others or about past or future events [12]. The Chinese version of the scale was revised by Dai et al. [31]. The scale consists of 10 items (e.g., “I worry that bad things may happen to me”). These items are assessed using a five-point Likert scale, ranging from 1 (does not apply to me at all) to 5 (extremely applies to me). The Cronbach's alphas of the scale were 0.901 at T1 and 0.881 at T2.

#### 2.2.5. The Automatic Thoughts Questionnaire (ATQ)

The ATQ was used to measure individuals' automatic negative thoughts related to depression and anxiety [32]. The Chinese version was developed by Pan et al. [33]. This study used a simplified version of the eight-item measure. Participants rated each item with a five-point Likert scale ranging from 1 (not at all) to 5 (almost every day). The greater the total score, the more frequently the individual has negative automatic thoughts. The Cronbach's alphas of the scale were 0.946 and 0.947 at T1 and T2, respectively.

### 2.3. Statistical Analysis

SPSS 23.0 was used for statistical analysis, and an independent samples *t* test was performed. R studio 4.3.0 was used for network analysis, which included five steps: network estimation, centrality estimation, network accuracy and stability estimation, network comparison, and cross-lagged prospective network analysis (CLPN).

#### 2.3.1. Network Estimation

To flexibly estimate the network structure and to construct a network model, we used the “estimate network” function within the “boot net” toolkit. This function allows for the incorporation of various R packages and network model frameworks [34]. This approach employs the least absolute shrinkage and selection operator regression (LASSO) method and the extended Bayesian information criterion to determine the most suitable regularization parameters for estimating Gaussian–Markov random fields. The regularization operations were primarily conducted using graphical LASSO [35] to obtain the regularization coefficients. Subsequently, the extended Bayesian method [36] was employed to model these coefficients. In total, 100 models were constructed. Finally, the model with the lowest EBIC value was selected, The hyperparameter*γ* was set to 0.5 to achieve a balance between sensitivity (i.e., elimination of true edges) and specificity (i.e., retention of false positive edges), thereby maximizing the likelihood of selecting true edges. In addition, we employed the “average layout” feature in the “q graph” toolkit to standardize the arrangement of diverse network models [37].

#### 2.3.2. Centrality Estimation

To measure each node's importance in the network, we computed the centrality index of each node using the “centrality plot” function in the “q graph” package. Because the nodes were positively intercorrelated, we chose strength, closeness, and betweenness as the centrality indicators [38]. Strength is represented by the value of the connection between nodes, taking into account their respective weights. Closeness is the sum of the distances among a specific node and all other nodes; it indicates the local structure of the node being studied and the extent of its involvement (or position) in the network. Betweenness refers to the extent to which a node is traversed by the shortest path between multiple nodes, indicating the node's capacity to adapt within the network.

#### 2.3.3. Stability Estimation

Random cluster sampling to select participants may yield data that is not representative, potentially impacting the estimated network model. Therefore, it is necessary to conduct sensitivity analysis to assess the accuracy and stability of edge weights and centrality indices [39]. The sensitivity analysis was conducted using the nonparametric bootstrapping technique in the “bootnet” package. This technique involves resampling the data with replacement, and the parameter estimates derived from these resampled samples are used to construct 95% confidence intervals (CIs). A constructed network with a narrower CI is considered more reliable than one with a wider confidence interval [40]. Whereas when absolute edge weights are used to calculate the centrality index, there may not be sufficient accuracy when using CIs alone to assess centrality stability. Hence, Epskamp et al. [34] proposed using a case-drop bootstrap to assess the precision of centrality index estimation. This procedure enhances the measurement of stability by analyzing the correlation between the estimated centralities in the original and subset samples and then calculating the correlation stability (CS) coefficient. Centrality indices are deemed stable if their strength remains relatively constant when a small subsample is taken, which involves randomly excluding 70% of the data from the original dataset. Furthermore, Epskamp et al. [34] suggested that the CS coefficient should exceed 0.5 and should not fall below 0.25. In the current study, we conducted 1,000 iterations of bootstrapping; the CS coefficient is 0.75.

#### 2.3.4. Network Comparison

We compared the during COVID-19 network and after the COVID-19 network using the network comparison. The permutation test method was used to evaluate the disparity in network structure between the two estimation models (T1 and T2) [41]. This function computes the invariance of global strength and edge weights to account for variations in global and local characteristics across different networks. Local variance pertains to the disparity in edge weight or centrality index for each node, which is indicative of alterations in the relationship between corresponding nodes across distinct networks. The global strength value represents the collective interconnectedness of the nodes in the network. A higher value of global strength indicates stronger connectivity among all the valuables among negative bias thinking, automatic negative thinking, symptom rumination, brooding, reflective pondering, depression, and anxiety [42].

#### 2.3.5. Cross-Lagged Prospective Network Analysis (CLPN)

We computed a directed CLPN from T1 to the follow-up T2 assessment (i.e., T1→T2) by using the “glmnet” package [43]. We calculated cross-lagged coefficients by using regression models that accounted for all other symptoms at T1 and then predicted symptoms at T2. To build this network, we used the LASSO penalty maximum likelihood approach, employed tenfold cross-validation to optimize the parameters, and eliminated small regression coefficients to mitigate the impact of spurious relationships on the conclusion. In graphic depictions of each network, symptoms are depicted as nodes, the direction of a cross-lagged effect is indicated by an arrow, and the thickness of the line corresponds to the strength of the association. We computed the centrality index for the CLPN network using the cross-lagged approach, specifically measuring the expected influence (IEI) for incoming connections and the expected influence (OEI) for outgoing connections. IEI quantifies the extent to which each node at T2 contributes to the overall variance in the network and the extent to which this contribution can be attributed to all nodes at T1. OEI refers to the degree to which a specific node at T1 can account for the variability observed in all nodes at T2 [25].

## 3. Results

### 3.1. Descriptive Analysis

Table 1 shows the descriptive statistics for the demographic variables at T1 and T2. At T1 (during the pandemic, *N* = 2,709), the prevalence rate of depression was 42.3%, while the prevalence rate of anxiety was 31.2%. At T2 (after the pandemic, *N* = 1,745), the prevalence rate of depression dropped to 40.5%, and the prevalence rate of anxiety rose to 33.5%. Because there were only 689 participants who were included in the analyses (those who submitted valid questionnaires at both T1 and T2), we tested whether there were demographic differences between this group at T1 and the full sample at T1 (*N* = 2,709). Independent samples *t* tests found no significant difference on any demographic variable, suggesting that the participants with matched data were representative of the larger group of participants who were recruited for the study.

We conducted paired samples *t* tests to compare T1 and T2 scores on the seven study variables. There were significant decreases in depressive symptoms and in brooding. There were significant increases in anxiety symptoms and negative attention bias. There was no significant change in symptom rumination, reflective pondering, or negative automatic thoughts. See Table 2.

### 3.2. Contemporaneous Networks

#### 3.2.1. Network Estimation

**Figure 3:**
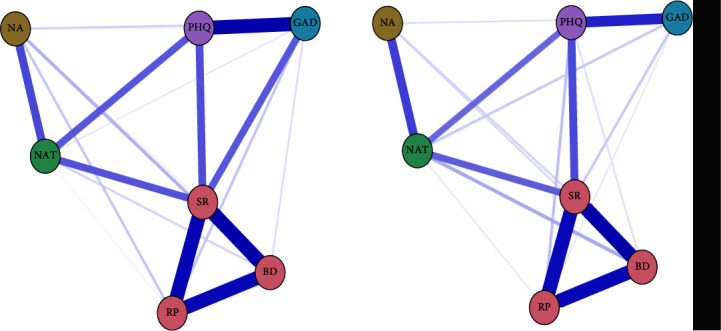
Contemporaneous network structure diagrams at T1 and at T2 (the left side of Figure 3 is the network diagram at T1, and the right side is the network diagram at T2. PHQ, depression; GAD, anxiety; SR, symptom rumination; BD, brooding; RP, reflective pondering; NA, negative attention bias; NAT, negative automatic thoughts).

**Figure 4:**
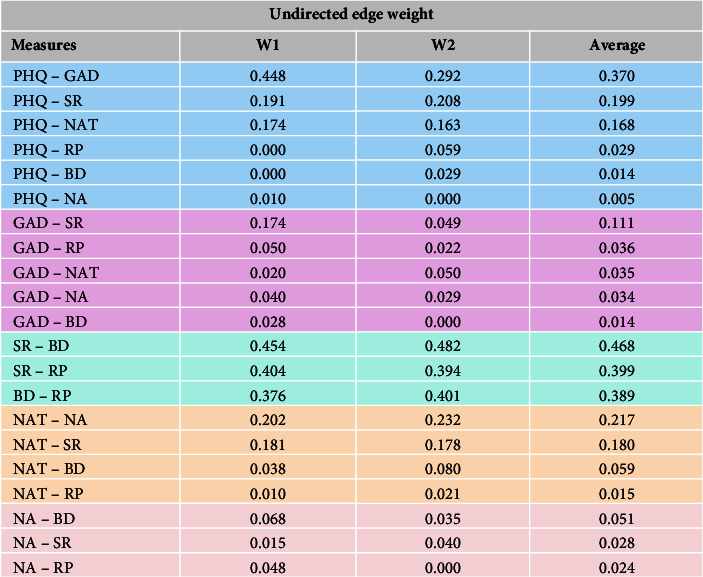
Undirected prediction table from T1 to T2 in the simultaneous network (PHQ, depression; GAD, anxiety; SR, symptom rumination; BD, brooding; RP, reflective pondering; NA, negative attention bias; NAT, negative automatic thoughts).

Figure 3 shows the constructed network. Seven variables should form a total of 21 edges. There were 19 nonzero edges in the T1 network and 18 nonzero edges in the T2 network. The sparsity of the T1 network was 0.9, while the sparsity of the T2 network was 0.86. In both networks, the edge weights between the three types of rumination (symptom rumination, brooding, and reflective pondering) were the highest. Outside this structure, the edge weights between depression and anxiety were the highest in both networks. The edge weights between these two variables (depression and anxiety) showed a significant decrease from T1 to T2.  Figure 4 shows the specific edge weight values.

#### 3.2.2. Centrality Estimations

**Figure 5:**
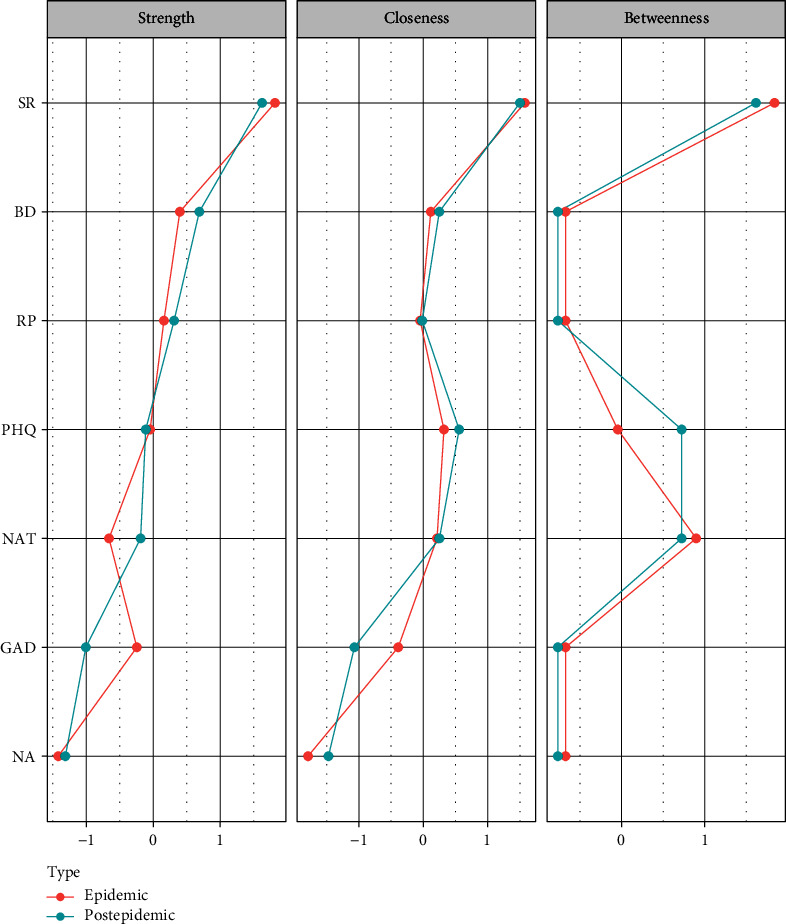
*Z*-score values of the three centrality indicators in each network: strength, closeness, and betweenness (PHQ, depression; GAD, anxiety; SR, symptom rumination; BD, brooding; RP, reflective pondering; NA, negative attention bias; NAT, negative automatic thoughts).

Centrality strength is the centrality of nodes in the entire comorbidity network.  Figure 5 shows that in both the T1 and T2 networks, the nodes with the three strongest indicators were all linked to “symptom rumination,” and the weakest nodes were all “negative attention bias.” The fact that this pattern was evident both at T1 and T2 reflects the influence of this symptom in the network stability across time. The order of strength from strong to weak was as follows: symptom rumination, brooding, reflective pondering, depression, negative automatic thinking, anxiety, and negative attention bias.

#### 3.2.3. Accuracy and Stability Estimation

**Figure 6:**
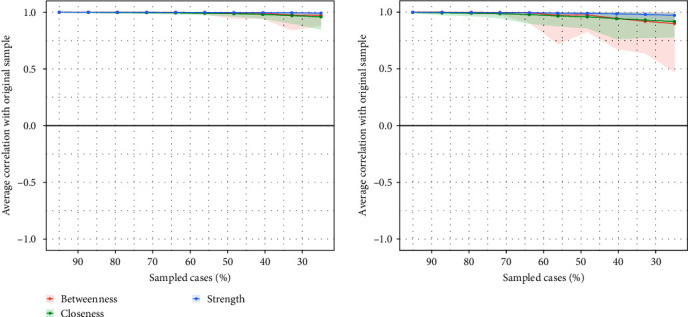
Stability of the estimated centrality index using the case-drop bootstrapping method (the left graph shows the stability test results at T1, and the right graph shows the stability test results at T2).

There was good stability and accuracy in the network structure. The bootstrapping method yielded a relatively narrow edge weight confidence interval (CI) that demonstrated acceptable accuracy.  Figure 6 shows the results of the centrality stability estimation process, which demonstrated good acceptability in both networks. At T2, the network betweenness index value was 0.672, while all other index values were 0.75.

#### 3.2.4. Network Comparison

We compared the T1 and T2 networks in terms of global and local structure. There was no significant difference in the overall strength of the two networks (*p*=0.127). However, there was a significant difference between T1 and T2 in edge weights (*p*  < 0.01). Specifically, there was a significant decrease in the edge weight between anxiety and symptom rumination (with a difference value of 0.18, *p*  < 0.05). Additionally, there was a significant increase in the edge weights between depression and reflective pondering, brooding, and negative automatic thinking, negative attention bias, and negative automatic thinking.

### 3.3. Temporal Networks

**Figure 7:**
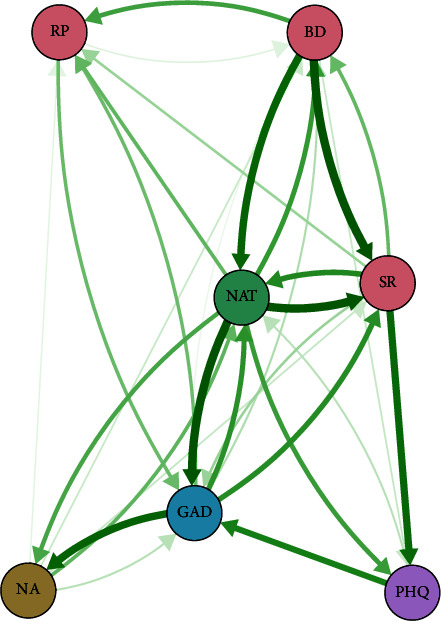
Time network structure diagram constructed based on all study variable (PHQ, depression; GAD, anxiety; SR, symptom rumination; BD, brooding; RP, reflective pondering; NA, negative attention bias; NAT, negative automatic thoughts).

**Figure 8:**
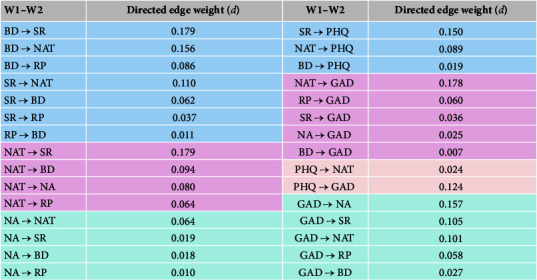
Directed prediction table from T1 to T2 in the temporal network.

Figure 7 shows the temporal networks, and Figure 8 shows the predictive relationships among variables in the temporal network. There were three types of problematic thinking that predicted later depressive symptoms, the strongest of which was symptom rumination (*d* = 0.15), followed by negative automatic thinking (*d* = 0.089) and brooding (*d* = 0.019). All five dimensions of problematic thinking predicted later anxiety symptoms, with negative automatic thinking (*d* = 0.178) being the strongest predictor.

From T1 to T2, depressive symptoms only predicted negative automatic thoughts, while anxiety predicted all five aspects of problematic thinking to varying degrees. Anxiety predicted the three dimensions of rumination with the following effect sizes: negative attention bias (*d* = 0.157), symptom rumination (*d* = 0.105), and negative automatic thinking (*d* = 0.101).

**Figure 9:**
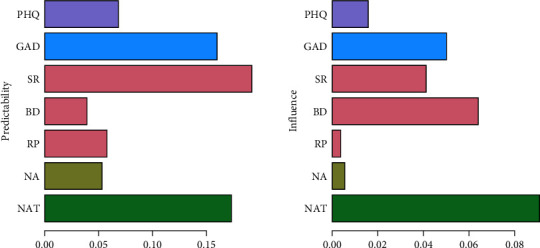
Prediction and influence as indicators of centrality in the temporal network structure diagram (PHQ, depression; GAD, anxiety; SR, symptom rumination; BD, brooding; RP, reflective pondering; NA, negative attention bias; NAT, negative automatic thoughts).

Surprisingly, depression predicted later anxiety, but anxiety did not predict later depression. There was a bidirectional predictive relationship among the three independent variables in the process of rumination, except for reflective pondering, which only predicted brooding. Furthermore, it is important to note that there existed a reciprocal predictive association between negative automatic thoughts and symptom rumination, with negative automatic thoughts exhibiting superior predictive capabilities for symptom rumination.  Figure 9 shows that the variable with high influence and low predictive power was brooding; the variable with high predictive power and low influence was symptom rumination; and the variable with high predictive power was negative automatic thinking.

## 4. Discussion

Earlier studies showed a significant rise in depression and anxiety symptoms during the pandemic compared to the prepandemic period [2, 44]. In the current study, we tested whether college students' depressive and anxiety symptoms during the pandemic lockdown had changed 6 months later, after lockdown. We found that depressive symptoms significantly decreased, while anxiety symptoms significantly increased, over this period; there was also an increase in brooding (a type of rumination) and a decrease in negative attention bias (a type of cognitive distortion). Cross-lagged prospective network analysis showed that cognitive distortion during lockdown (especially symptom rumination) predicted psychological distress after lockdown; psychological distress during lockdown (especially anxiety symptoms) predicted rumination after lockdown. Surprisingly, anxiety symptoms predicted later depressive symptoms, but depressive symptoms did not predict later anxiety symptoms. This is the first study to compare distress measured during and after lockdown for the pandemic. The results have implications for cognitive models of depression and anxiety, and they have potential application for providing support services to college students during and after periods of crisis.

We approached the analyses in two ways. First, we compared the college students' self-reported depressive symptoms, anxiety symptoms, rumination, and cognitive distortion during lockdown to scores on the same measures after lockdown, 6 months later. The results showed that compared to T1, scores for depressive symptoms were significantly lower at T2, and scores for anxiety symptoms were significantly higher at T2. Brooding, a type of rumination, increased; negative attention bias, a type of cognitive distortion, decreased. The findings lead to the question of how these changes in anxiety symptoms, depressive symptoms, rumination, and cognitive distortion are related. Are there longitudinal connections among them, either unidirectional or bidirectional?

The second approach to the analyses shed light on these questions by focusing not on mean T1–T2 differences but on the associations among variables over time. Network analysis provided a way to create maps of the interrelations among all the study variables at T1 and at T2 and longitudinally from T1 to T2. The three maps were similar. Certain variables (nodes) stood out in terms of the number of connections to other variables and the strength of these connections; the details are described below.

The relationships among all the variables from T1 to T2 in the network showed that the correlation between depressive and anxiety symptoms far outweighed the other relationships, consistent with established evidence of the comorbidity of depression and anxiety [45, 46, 47]. However, we were surprised to find that whereas depressive symptoms predicted later anxiety symptoms, the reverse was not true: anxiety symptoms did not predict later depressive symptoms. It has been proposed that anxiety is a secondary symptom of depression [48], although it has also been proposed that anxiety and depression mutually influence each other equally [46].

Although anxiety symptoms and depressive symptoms were comorbid, the constructed networks showed that each had unique associations with the cognitive distortions and dimensions of rumination that we assessed. This suggests that the two forms of distress may have distinct internal formation mechanisms. First, we consider the bidirectional, longitudinal among between depressive symptoms and cognitive distortion. We then consider these same links as they involve anxiety rather than depression.

The cognitive model of depression is based on the premise that automatic negative thinking is the most critical intermediate process leading to depression [49]. However, in our study, the network analysis showed that not only in T1 but also in T2, the link between depressive symptoms and another cognitive distortion, namely, negative attentional bias, was also extremely weak. This finding is consistent with those of a classical meta-analysis [50]. On the other hand, experimental research on attentional bias correction training for patients with depression showed that reducing patients' negative attentional bias helped reduce depressive symptoms [13].

However, while negative attention bias had a weak correlation with depression in this study, it had a strong correlation with dimensions of rumination. Researchers have highlighted the significance of rumination in the development of depression [51]. An intervention also showed a connection between rumination and depression. Watkins et al. [52] attempted to reduce depression by reducing general rumination using a form of cognitive behavioral therapy (RFCBT) dubbed RFCBT. The test of the therapy used a multiple baseline design to evaluate 14 depressed patients who received 12 consecutive sessions of RFCBT. Average depressive symptoms were effectively relieved at the end of the experiment.

Like depressive symptoms, anxiety symptoms showed bidirectional relationships with cognitive distortion. Anxiety was found to be a stronger predictor of later negative attentional bias than expected. According to existing cognitive models of anxiety, negative attentional bias is an important factor in anxiety maintenance [53, 54].

The findings of the current study demonstrates the core node of symptom rumination. The highest correlation with anxiety was symptom rumination, much higher than the correlations involving the other dimensions. Furthermore, symptom rumination had a stronger correlation with depression and anxiety than the other dimensions of rumination and the two types of cognitive distortion. Of the five patterns of problematic thinking, the strongest indicator of depression was symptom rumination, followed by negative automatic thoughts. While research on cognitive behavioral therapy (CBT) has consistently shown the significance of negative automatic thoughts in depression [49, 55], the results of this study suggest that symptom rumination may hold greater importance. This finding also suggests that the notable influence of rumination on depression may primarily stem from symptom rumination. Rumination about symptoms was the most predictive depression, even exceeding the influence of the more commonly researched effects of negative automatic thinking. Furthermore, compared to symptom rumination, negative automatic thoughts were more predictive of anxiety. This implies that future treatment of clinical symptoms of anxiety should prioritize negative automatic thoughts over rumination. Anxiety was a better predictor of symptom rumination than it was of negative attention bias. Therefore, we assert that symptom rumination may play an important role in the coexistence of depression and anxiety, and that it should be studied further.

This research has several limitations. First, we studied a community sample of college students, and the results might be different in a sample with clinically significant levels of anxiety and depression. Second this study did not distinguish various subcategories of depressive and anxiety symptoms. Bai et al. [45], for example, investigated problematic patterns of thinking in greater depth by including subcategories of depression and anxiety as nodes in network analysis. Third, the college students in our sample were all from South China, and it remains to be seen if the results will generalize to other parts of the country. Fourth, this was a cohort study rather than a longitudinal study. In addition, our sample size was reduced because we only included participants who provided valid data at both time points. Finally, the variables examined in this research were assessed through self-report questionnaires, which are prone to bias due to the effects of subjective recall, social desirability, and social stigma. Interviews would be a helpful complement in assessing psychological distress.

## 5. Conclusion

Several studies have shown that college students' psychological distress before the COVID-19 pandemic increased during the pandemic. This is the first study to compare college students' distress during and after the pandemic. Relative to their functioning during lockdown, students' anxiety increased, and their depression decreased, after lockdown. Patterns of rumination and cognitive distortion helped explain these changes. The findings challenge Beck's cognitive model by pointing to symptom rumination as a key predictor of later depression, and negative automatic thoughts as a key predictor of later anxiety. The results have potential application for helping students who continue to experience distress after the pandemic.

## Figures and Tables

**Table 1:** Demographic characteristics.

Variable	T1 (*N* = 2,709)		T2 (*N* = 689)	
Gender, *n* (%)				
Men	611	(22.49)	127	(18.43)
Women	2,106	(77.51)	562	(81.57)
Age, M (IQR)	19.81	(1.09)	19.72	(0.96)
Ethnicity, *n* (%)				
Han	2,661	(97.94)	678	(98.40)
Minority (e.g., Zhuang)	56	(2.06)	11	(1.60)
Place of residence, *n* (%)				
Urban	1,050	(38.65)	265	(38.46)
Rural	1667	(61.35)	424	(61.54)
Economic status, *n* (%)				
Much better	41	(2.51)	11	(1.60)
Better	250	(9.20)	60	(8.71)
Similar	1,428	(52.56)	373	(54.14)
Worse	778	(28.63)	191	(27.72)
Much worse	220	(8.10)	54	(7.84)
Whether the father worked outside the home for a long time, *n* (%)				
Yes	798	(29.37)	213	(30.91)
No	1,919	(70.63)	476	(69.09)
Whether the mother worked outside the home for a long time, *n* (%)				
Yes	534	(19.65)	142	(20.61)
No	2,183	(80.35)	547	(79.39)
Only child, *n* (%)				
Yes	367	(13.51)	88	(12.77)
No	2,350	(86.49)	601	(87.23)
History of mental illness, *n* (%)				
Yes	30	(1.10)	5	(0.73)
No	2,687	(98.90)	684	(99.27)
History of smoking, *n* (%)				
Never	2,537	(93.37)	654	(94.92)
Past	95	(3.50)	16	(2.32)
Present	85	(3.13)	19	(2.76)
History of alcohol consumption, *n* (%)				
Never	2,171	(79.90)	563	(81.71)
Past	180	(6.63)	40	(5.81)
Present	366	(13.47)	86	(12.48)
History of romantic relationships, *n* (%)				
Never	1,324	(48.73)	356	(51.67)
Previous	745	(27.42)	191	(27.72)
Present	648	(23.85)	142	(20.61)
Somatic symptoms, *n* (%)				
Yes	787	(28.97)	213	(30.91)
No	1,930	(71.03)	476	(69.09)

**Table 2:** Results of paired samples *t* tests comparing T1 and T2 scores on the of study variables (*N* = 689).

Variable	*t*	*p*
PHQ	2.142	0.032^*∗*^
GAD	−14.641	<0.01^*∗∗*^
SR	1.539	0.124
BD	2.016	0.044^*∗*^
RP	1.174	0.240
NA	−1.998	0.046^*∗*^
NAT	−1.79	0.074^*∗*^

*⁣* ^*∗*^ *p* < 0.05, *⁣*^*∗∗*^*p* < 0.01. T1 = Time 1, during pandemic; T215 = Time 2, after pandemic. PHQ, depression symptoms; GAD, anxiety symptoms; SR, symptom rumination; BD, brooding; RP, reflective pondering; NA, negative attention bias; NAT, negative automatic thoughts.

## Data Availability

The datasets generated during and/or analyzed during the current study are available from the corresponding author on reasonable request.
